# Supplementary material for: The interactome of intact mitochondria by cross-linking mass spectrometry provides evidence for coexisting respiratory supercomplexes
Source: Mol Cell Proteomics. 2017 Dec 8;17(2):216–32. doi: 10.1074/mcp.RA117.000470 (PMC5795388; doi:10.1074/mcp.RA117.000470)
Supplement: Supplemental Data [file supp_RA117.000470_133922_0_supp_23978_fzffwf.zip › spectra_annotation/mito_spectra_annotation/454-1-6-1-2-1.pdf]

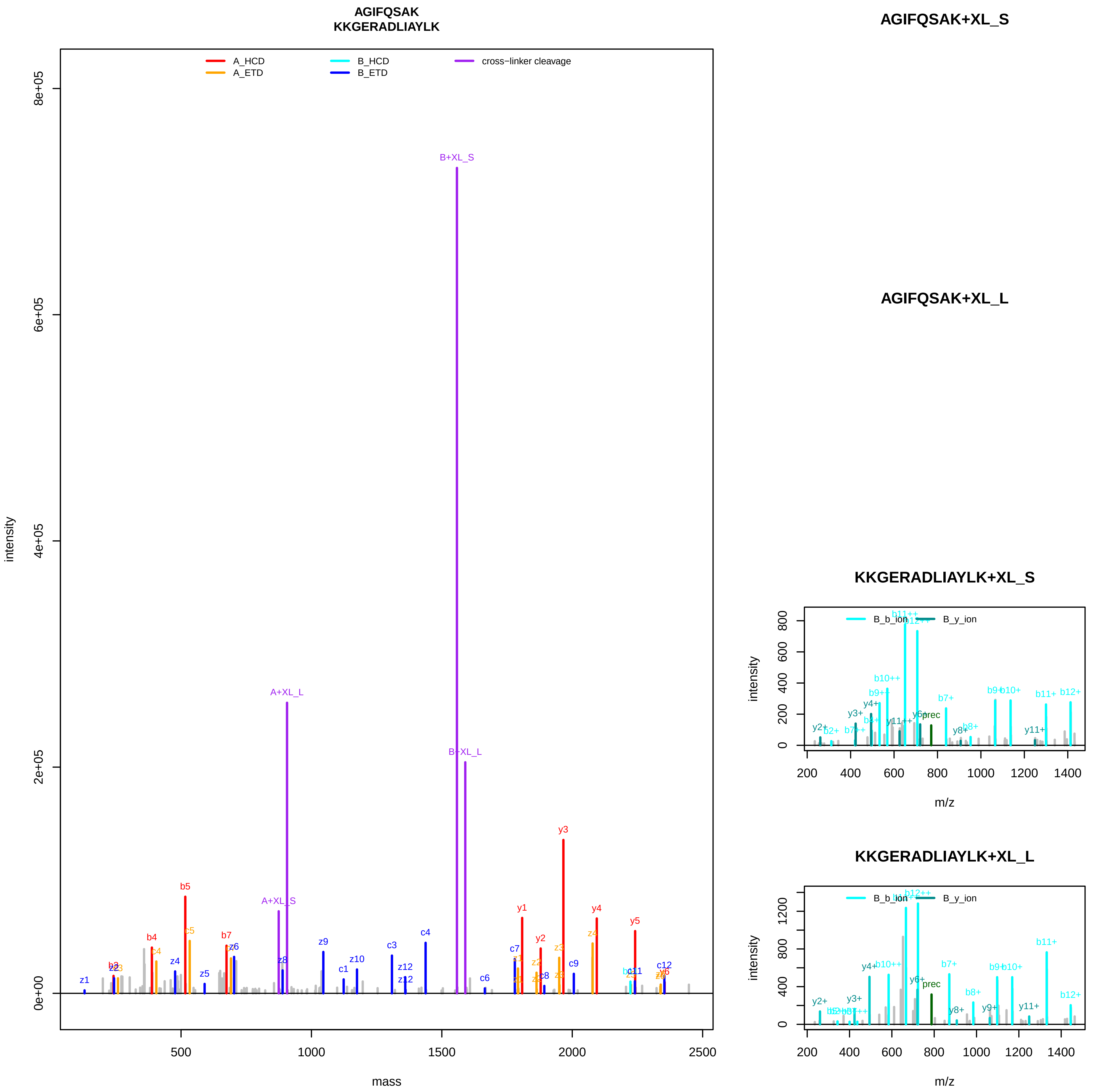
AGIFQSAK
KKGERADLIAYLK
A_HCD
A_ETD
B_HCD
B_ETD
cross−linker cleavage
intensity
mass
0e+00
2e+05
4e+05
6e+05
8e+05
500
1000
1500
2000
2500
B+XL_S
A+XL_L
B+XL_L
A+XL_S
b3
b4
b5
b7
c4
c5
z1
z2
z3
z4
z5
z6
z8
z9
z10
z12
c1
c3
c4
c6
c7
c8
c9
c11
c12
y1
y2
y3
y4
y5
y6
AGIFQSAK+XL_S
AGIFQSAK+XL_L
KKGERADLIAYLK+XL_S
B_b_ion
B_y_ion
intensity
m/z
0
200
400
600
800
1000
1200
1400
b9++
b10++
b11++
b12++
b7+
b9+
b10+
b11+
b12+
y2+
y3+
y4+
y6+
y8+
y11+
y11++
prec
KKGERADLIAYLK+XL_L
B_b_ion
B_y_ion
intensity
m/z
0
400
800
1200
200
400
600
800
1000
1200
1400
b10++
b12++
b7+
b8+
b9+
b10+
b11+
b12+
y2+
y3+
y4+
y6+
y8+
y9+
y11+
prec
